# Multiplexed targeting of miRNA-210 in stem cell-derived extracellular vesicles promotes selective regeneration in ischemic hearts

**DOI:** 10.1038/s12276-021-00584-0

**Published:** 2021-04-20

**Authors:** Byeong-Wook Song, Chang Youn Lee, Ran Kim, Won Jung Kim, Hee Won Lee, Min Young Lee, Jongmin Kim, Jee-Yeong Jeong, Woochul Chang

**Affiliations:** 1 Institute for Bio-Medical Convergence, Catholic Kwandong University International St. Mary’s Hospital, Incheon, Republic of Korea; 2 Department of Integrated Omics for Biomedical Sciences, Graduate School, Yonsei University, Seoul, Republic of Korea; 3 Department of Biology Education, College of Education, Pusan National University, Busan, Republic of Korea; 4 Department of Molecular Physiology, College of Pharmacy, Kyungpook National University, Daegu, Republic of Korea; 5 Department of Life Systems, Sookmyung Women’s University, Seoul, Republic of Korea; 6 Department of Biochemistry, Kosin University College of Medicine, Busan, Republic of Korea

**Keywords:** Myocardial infarction, Stem-cell research

## Abstract

Extracellular vesicles (EVs) are cell derivatives containing diverse cellular molecules, have various physiological properties and are also present in stem cells used for regenerative therapy. We selected a “multiplexed target” that demonstrates multiple effects on various cardiovascular cells, while functioning as a cargo of EVs. We screened various microRNAs (miRs) and identified miR-210 as a candidate target for survival and angiogenic function. We confirmed the cellular and biological functions of EV-210 (EVs derived from ASC^miR-210^) secreted from adipose-derived stem cells (ASCs) transfected with miR-210 (ASC^miR-210^). Under hypoxic conditions, we observed that ASC^miR-210^ inhibits apoptosis by modulating protein tyrosine phosphatase 1B (PTP1B) and death-associated protein kinase 1 (DAPK1). In hypoxic endothelial cells, EV-210 exerted its angiogenic capacity by inhibiting Ephrin A (EFNA3). Furthermore, EV-210 enhanced cell survival under the control of PTP1B and induced antiapoptotic effects in hypoxic H9c2 cells. In cardiac fibroblasts, the fibrotic ratio was reduced after exposure to EV-210, but EVs derived from ASC^miR-210^ did not communicate with fibroblasts. Finally, we observed the functional restoration of the ischemia/reperfusion-injured heart by maintaining the intercommunication of EVs and cardiovascular cells derived from ASC^miR-210^. These results suggest that the multiplexed target with ASC^miR-210^ is a useful tool for cardiovascular regeneration.

## Heart disease: Boosting microRNA activity may restore heart function

A therapy designed to enhance the activity of a small regulatory RNA molecule could prompt the restoration of cellular communication and improve heart function during heart disease. Heart cells struggle to function and regenerate under the reduced oxygen conditions caused by narrowed or blocked blood vessels. Stem cell-derived therapies may restore damaged heart tissues, and can be designed to secrete specific factors that boost regeneration. To identify factors that may improve heart recovery chances, Woochul Chang at Pusan National University, Busan, Korea and co-workers examined stem cell-derived extracellular vesicles (EVs), cellular membrane sacs that carry proteins and RNAs to surrounding tissues and boost intercellular communication. The team identified microRNA-210 as a key factor that inhibits heart cell death, reduces fibrosis, and enhances regeneration of heart cell populations. EVs secreting microRNA-210 may prove a useful therapeutic tool.

## Introduction

Cardiovascular diseases, including heart attacks and strokes, result in impairment of circulating organs and a subsequently shortened life span. Coronary heart diseases (CHDs) are the most common type, inducing arrhythmias and myocardial infarction (MI)^1^. Unlike lower vertebrates, adult mammals have difficulty in natural myocardial regeneration, and many therapeutic approaches have been researched for heart regeneration and repair^2,3^. The heart comprises cardiac myocytes and nonmyocytes, including fibroblasts, endothelial cells, and vascular smooth muscle cells. Furthermore, transient cellular constituents of the heart include lymphocytes, mast cells, and macrophages, which can affect cardiac function^4^. For normal cardiac function, cell populations of the heart are harmonized to maintain intercellular homeostasis and function as cellular control networks, including autocrine, paracrine, and cell–cell interactions. However, in the diseased state, this innate condition cannot maintain cardiomyocyte homeostasis and ultimately fails to restore the heart performance^5^.

Stem cells used to restore damaged hearts and wounded vessels are known to induce a multifaceted regenerative response through paracrine action by secreting various growth factors, that stimulate the survival and repair of host myocardium and recruit endogenous stem cells^5,6^. Various stem cell types from the embryonic to the adult stage have been considered as treatments for the regenerative process of cardiovascular diseases. Although stem cells are isolated from a single organ, they have mixed cellular phenotypes that result in heterogeneous outcomes or cause undesirable results, such as carcinogenic effects^7,8^. Therefore, a method has been proposed to induce appropriate treatment of the damaged heart by changing the phenotype of cells or differentiating them into target cells through proper modulation of stem cells^9–11^. However, cell contamination, cell death, and immune rejection significantly limit the clinical use of stem cells^12^. Alternative therapies such as cell-derived products have been proposed to address the disadvantages of cardiovascular disease treatment using stem cells.

Extracellular vesicles (EVs) are derivatives of cells; they are small extracellular membrane fragments that contain various types of cellular information and notable physiological properties. EVs predominantly consist of exosomes, small homogeneous vesicles, and microvesicles. EVs have a more heterogeneous dimensional profile generated due to direct extrusion of the cytoplasmic membrane^13^, and contain numerous lipids, proteins, and RNAs, including noncoding RNAs. In the process of cardiovascular disease and repair, the composition and internal cargo of EVs play a role in cell-to-cell communication. Notably, EVs may be a major factor in paracrine effects, including humoral stimulation of endogenous regeneration and preservation of pre-existing cells^14^. From the viewpoint of cell therapy, stem cell EVs are exogenous factors that have the capacity to stimulate myocardial regeneration and cardioprotective effects. Since cardiovascular function is dependent on cardiovascular cell interactions and communication, stem cell-derived EVs can increase several factors that are useful for survival and functional improvement. Despite this important mechanism, few studies have researched EVs derived from stem cells with specific traits regulating myocardial cells and nonmyocardial cells within the diseased heart^15^. This study demonstrates that miR-210 is a key regulator in ischemic myocardial cells and adipose-derived stem cells (ASCs), and that ASC-derived exosomes (EV-210) overexpressing miR-210 selectively affect myocardial/nonmyocardial cells.

## Materials and methods

### Culture of hASCs

Human adipose-derived stem cells (hASCs) were purchased from Invitrogen (Waltham, MA, USA). hASCs were cultured according to the manufacturer’s instructions. We used Dulbecco’s modified Eagle’s medium (DMEM; Gibco, Waltham, MA, USA) containing 10% fetal bovine serum (FBS; Gibco) and 1% antibiotics (Gibco). The media were changed every 3 days, and cells from passages 7 to 10 were used for the experiments.

### Isolation of hASC-derived EVs

hASCs were incubated for 1 day after seeding in a 100 mm dish (1 × 10^6^ cells/dish), washed twice with PBS, and then cultured with 10 mL of DMEM without FBS for 24 h. After 24 h of incubation, the medium was collected and centrifuged at 3000 × *g* for 15 min at 4 °C, and then, only the supernatant was transferred to a new tube. The supernatant was filtered through a 0.22 mm PVDF filter (Millipore, Burlington, MA, USA). An appropriate volume of ExoQuick-TC™ Exosome Precipitation Solution (System Biosciences, Palo Alto, CA, USA) was added to the filtered culture medium and mixed well by inverting. After refrigeration for 12 h, the mixture was centrifuged at 1500×*g* for 30 min, and all supernatant was removed by aspiration. EV pellets were resuspended in 100 µL of PBS (phosphate buffered saline; Gibco) and stored at −80 °C for further use.

### In vitro ischemic condition

For induction of hypoxia, 80% confluent hASCs were plated in cell culture dishes. The serum-free media were degassed and then exposed in a hypoxic chamber (Thermo Fisher Scientific, Waltham, MA, USA) maintained below 5% CO_2_, 5% H_2_, and 1% O_2_ at 37 °C. H9c2 cells were harvested after an 18 h incubation period and incubated with TRIzol Reagent (Life Technologies, Frederick, MD, USA) for quantification of miR expression.

### Ischemia-reperfusion injury and cell transplantation

All experimental procedures for animal studies were approved by the Committee for the Care and Use of Laboratory Animals of Catholic Kwandong University College of Medicine (CKU01-2015-003-1), and performed in accordance with the Committee’s Guidelines and Regulations for Animal Care. Seven-week-old male Sprague-Dawley rats (220 ± 30 g) were used for in vivo experiments. After anesthetization via intraperitoneal injection of zoletil (30 mg/kg) and xylazine (10 mg/kg), rats were ventilated via trachea using a ventilator (Harvard Apparatus, Holliston, MA, USA) and then subjected to surgically induced MI, followed by cell transplantation. MI was produced by surgical occlusion of the left anterior descending coronary artery by ligation using a 7-0 Prolene suture. The rats were randomized into five groups (normal, IR control, IR + ASC, IR + ASC^miR-210^, and IR + ASC^miR-210^ GW4869). GW4869 is a potent inhibitor that blocks exosomes. For cell transplantation, ASCs (stained with PKH67) were suspended in 30 µL of PBS (1 × 10^6^ cells), and transplanted into the viable myocardium bordering the infarction at three injection sites using an insulin syringe (BD Ultra-Fine II, 0.3 mL) with a 30-gauge needle. For internalization of EV-210 by cardiac muscle cells, miR-210-conjugated FAM^TM^ (fluorescein amidite) dye or miR-210-conjugated Cy-3 (cyanine 3; Cosmogenetech, South Korea) was diluted in Opti-MEM at the working concentration. The mixture was blended with diluted Lipofectamine RNAiMAX reagent (1:1 ratio; Thermo Fisher, Hudson, NH, USA) for 5 min at room temperature. Fluorescent miR-lipid complexes were poured onto ASCs, and then, labeled ASCs were used for transplantation with the same number of cells. To determine whether cell transplantation could have a therapeutic effect after MI, we performed trichrome staining at 1 week after MI and cell transplantation.

### Left ventricular catheterization

Left ventricular catheterization was performed 3 weeks after infarction to assess hemodynamics. A Millar Mikro-tip 2F pressure-volume transducer (model SPR-838, Millar instruments, Houston, TX, USA) was introduced into the left ventricle via the right carotid artery under anesthesia. All data were analyzed offline with PVAN 3.5 software (Millar instruments).

### Statistical analysis

The data are expressed as the mean ± standard error of the mean of at least three independent experiments. Comparisons between more than two groups were performed by one-way analysis of variance using Bonferroni’s correction *p* < .05 was considered significant.

## Results

### MiR-210 plays an important role in hypoxic cardiomyocytes and ischemic myocardium

**Fig. 1:**
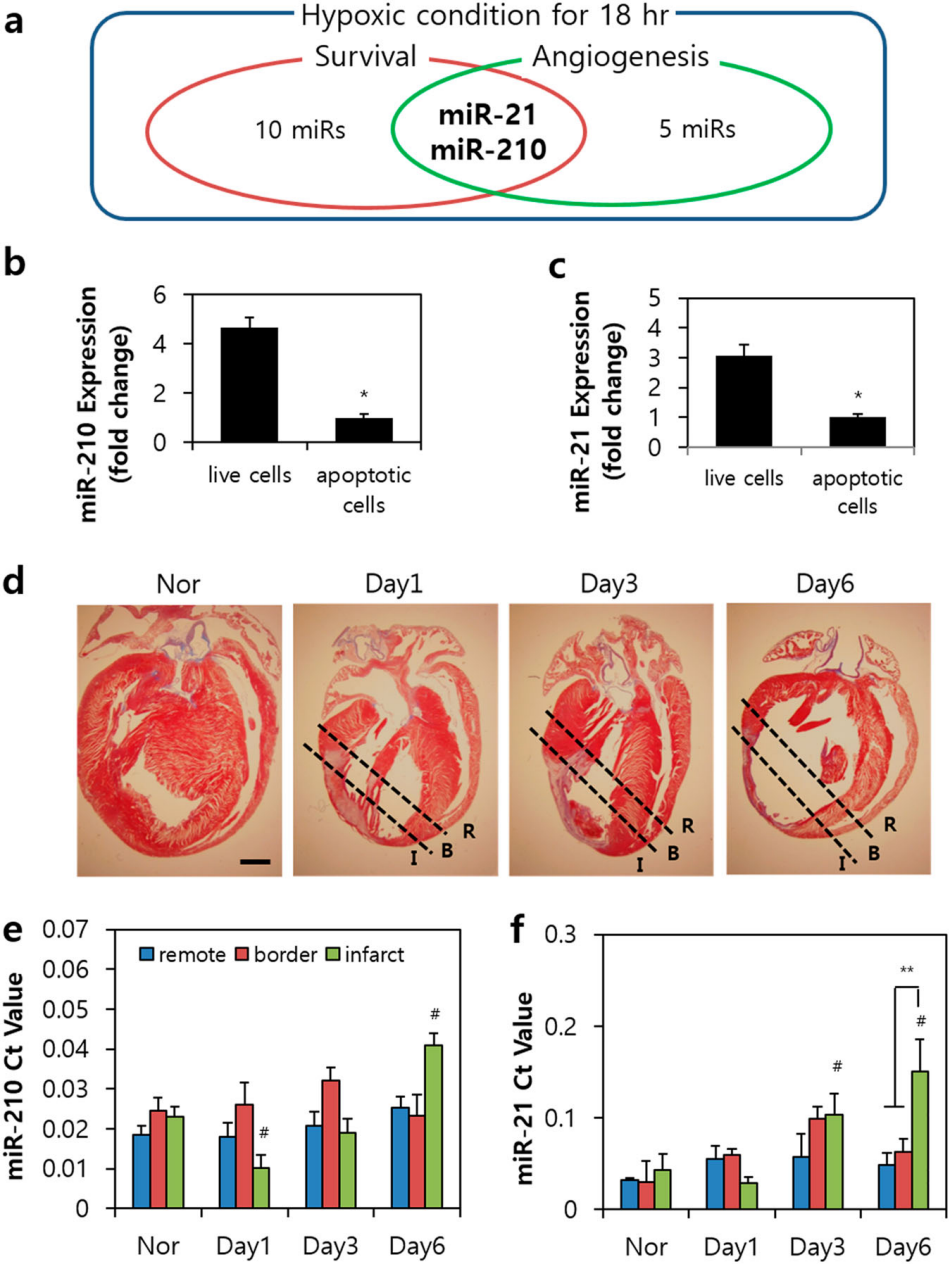
MiR-210 and miR-21 are involved in cardiac regeneration after ischemia. **a** Venn diagram of the miRNAs targeting survival-mediated molecules (red circle), miRNAs targeting angiogenesis-mediated molecules (green circle), and miRNAs regulated in hypoxic H9c2 cells for 18 h (blue circle). **b** Expression of miR-210 between live and apoptotic H9c2 cells. **c** Expression of miR-21 between live and apoptotic H9c2 cells. **d** Masson’s trichrome staining in normal heart and day 1, day 3, and day 6 rat heart sections after myocardial infarction. **e** Mean Ct values of miRNA-210 expression in three regions of the myocardium. **f** Mean Ct values of miRNA-21 expression in three regions of the myocardium. I infarct, B border-zone; R remote myocardium. Scale bars: 3 μm. *P < 0.05 vs. live cells, ^#^*P* < .0.5 vs. normal infarct, **P < 0.05. All values are the mean ± s.d. Statistical significance was assessed by one-way ANOVA.

The endogenous regeneration of cardiomyocytes can be induced by exposure to systemic hypoxia^16^. This phenomenon can result in significant functional recovery following MI. To determine the factors that play an important role in hypoxia, we performed functional screening of microRNAs (miRs) that control specific functions. We first analyzed the expression levels of selected miRs that are involved in the survival and angiogenesis of H9c2 cells under hypoxic conditions for 18 h (Supplementary Fig. 1a). MiR-210 and miR-21 showed lower expression levels than those in the control group (=1 a.u.) among 13 miRs regulating survival-related and angiogenesis-related proteins (Fig. 1a). After 18 h of incubation under hypoxic conditions, comparison of miR expression between live and apoptotic H9c2 cells revealed a 0.21-fold difference in miR-210 expression (Fig. 1b) and a 0.33-fold difference in miR-21 expression (Fig. 1c). Ischemia and reperfusion injuries (I/R) were created by left anterior descending artery (LAD) ligation in 8-week-old rats to evaluate the temporal expression of endogenous miRs under conditions of stress. To identify alterations of the two miRs under consideration, we compared miR-210 and miR-21 levels at the infarcted and noninfarcted (remote) margins of the myocardium after day 6 of I/R (Fig. 1d). MiR-21 showed a sharp increase in expression at the infarct margins from day 3 of the injury, whereas the expression of miR-210 increased gradually until day 6 in the I/R-injured heart (Fig. 1e, f). No other specific miR expression changes were observed in other regions.

This study was undertaken to specifically deliver miRs from EVs derived from ASCs to the heart. Second, we detected the expression profiles of 13 miRs in EVs from normal ASCs to measure the general expression levels of specific miRs before transfection and to select miRs associated with very few stem cells (Supplementary Fig. 1b). MiR-210 is present in the heart and is regenerated after injury but is lower in the early stage of I/R; it is therefore considered to be a suitable candidate, in contrast to miR-486, miR-31, miR-21, and miR-126, which are expressed above the reference value.

### EVs containing miR-210 regulate cardiovascular cells under ischemic conditions

Recent studies have shown that EVs secreted by stem cells contribute to their paracrine effects^14,17,18^. Considering previous results that EVs from transplanted stem cells influence cardiovascular disease via their paracrine effects, we hypothesized that EVs derived from miR-transfected ASCs would have specific therapeutic potential.

To precisely characterize EVs, we isolated extracellular vesicular fractions from ASCs as described previously^19^. Phase-contrast electron micrographs of ASCs transfected with miR-210 revealed smaller vesicles of EVs (Supplementary Fig. 2a left); we further isolated and collected EVs with a size of approximately 60–200 nm (Supplementary Fig. 2a right). Immunoblot analysis revealed that CD63 and CD81 (two members of the tetraspanin family and known hallmarks of EVs^20^) and CD44 (mesenchymal stem cell surface marker^21^) were present in the cell lysate. In contrast, the cellular proteins cytochrome c and β-actin were also detected (Supplementary Fig. 2b).

**Fig. 2:**
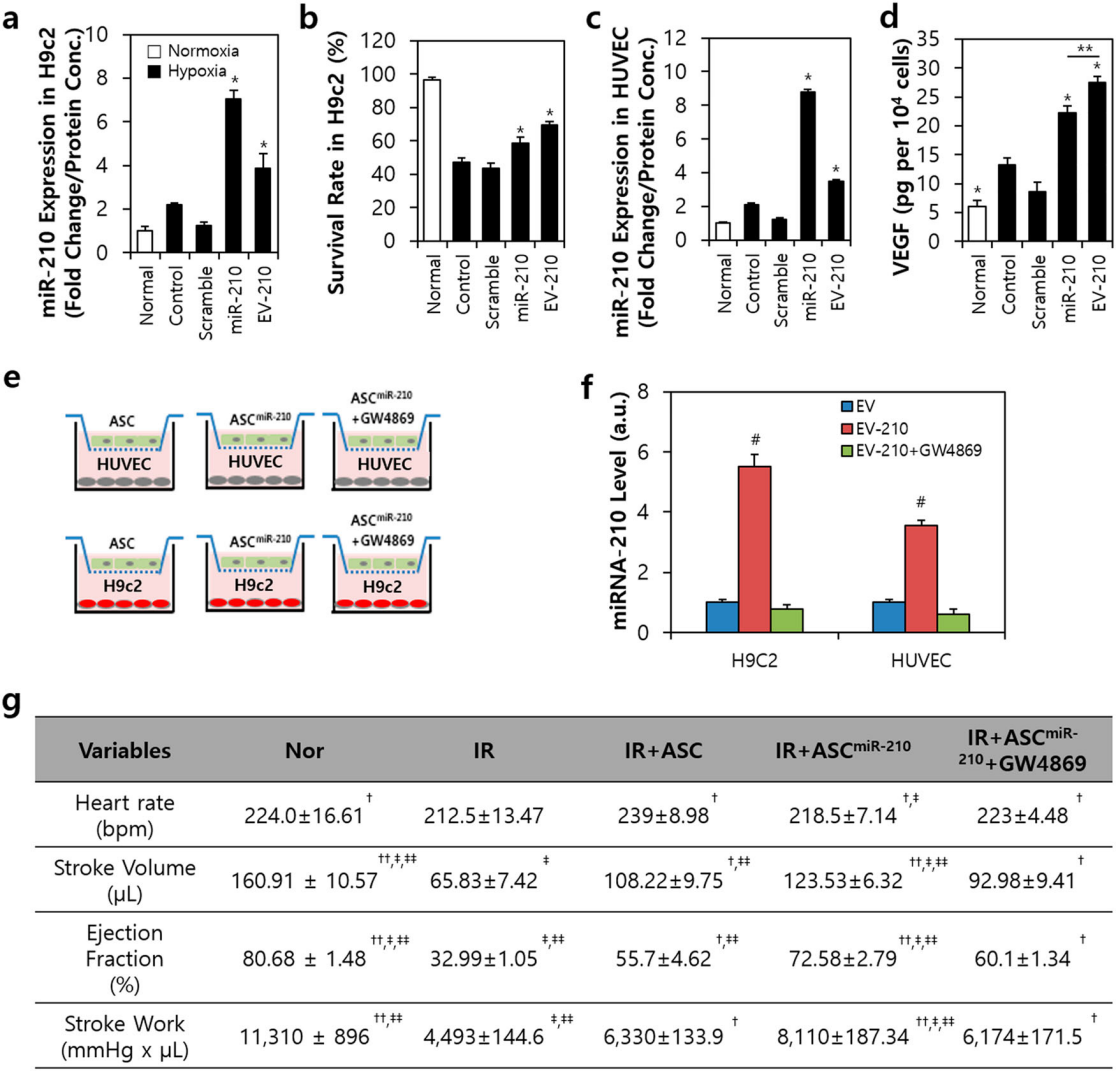
Exosomes derived from ASCs transfected with miR-210 participate in the regulation of cardiovascular cells. **a** Expression of miR-210 in H9c2 cells treated with miR-210 or EV-210. **b** Survival rate of H9c2 cells after miR-210 or EV-210 treatment. **c** Expression of miR-210 in human umbilical vein endothelial cells (HUVECs) treated with miR-210 or EV-210. **d** Secretion of VEGF after miR-210 or EV-210 treatment. **e** EV permeable system for ASC^miR-210^ cocultured with cardiovascular cells (HUVECs or H9c2 cells). **f** Expression level of miRNA-210 in H9c2 cells or HUVECs cultured in an exosome-permeable system. **g** Measurement of LV function by using a Millar catheter after ischemic-reperfusion (I/R) and immediate ASC treatment. ASC adipose-derived stem cell, ASC^miR-210^ miR-210-transfected ASCs, EV-210 extracellular vesicles derived from miR-210-transfected ASCs, HUVECs human umbilical vein endothelial cells, VEGF vascular endothelial growth factor, GW4869 exosome generation blocker, IR ischemia-reperfusion. Statistical analysis: **a**–**f** *P < .05 vs. the control, **P < 0.05, ^#^*P* < 0.05 vs. EV. **g** ^†^*P* < 0.05 vs. the IR group, ^††^*P* < 0.05 vs. the IR group, ^‡^*P* < 0.05 vs. IR + ASC, ^‡‡^*P* < .05 vs. IR + ASC^miR-210^ + GW4869. Statistical significance was assessed by one-way ANOVA.

We observed that the miR-210-transfected H9c2 cells overexpressed miR-210 by 3.6-fold compared to the hypoxic cells; furthermore, the EV-210-treated H9c2 cells expressed increases up to 1.9-fold (Fig. 2a). Moreover, the survival rate of H9c2 cells increased by 11% in the EV-210-treated group compared to the miR-210-treated group (Fig. 2b). In human umbilical vein endothelial cells (HUVECs), the expression of miR-210 was increased in the miR-210-transfected HUVECs (4.5-fold) and EV-treated HUVECs (1.9-fold) compared to the control cells (Fig. 2c). Exposure to miR-210 increased the expression of vascular endothelial growth factor (VEGF) to 169% in the miR-210 group (22 pg) and 211% in the EV-210 group (27.5 pg) compared to the control group (13 pg) (Fig. 2d). The 24 h indirect exposure procedure (i.e., the EV permeable system) of cardiovascular cells grown on ASCs, miR-210-transfected ASCs (ASC^miR-210^), or ASC^miR-210^ cotreated with GW4869 was modified from a previous study (Fig. 2e)^22^. GW4989, a neutral sphingomyelinase inhibitor, is a widely used pharmacological agent that blocks exosome generation^23^. We observed that after GW4869 treatment, the expression level of miR-210 decreased by 86% in H9c2 cells and 81% in HUVECs compared to that of the EV-210 group (Fig. 2f).

To determine the effect of EV-210, we performed echocardiography to assess cardiac function 3 weeks after I/R injury and immediate treatment in vivo (Fig. 2g). Compared to those of the I/R group, the stroke volume (SV), ejection fraction (EF) and SV of the ASC^miR-210^ group were increased 2-fold; however, in the group exposed to GW4869, cardiac function was almost lost. The cardiac function level of the ASC^miR-210^ group was 76.7% for SV, 89.9% for EF, and 71.7% for SW compared to that of the normal group. In contrast, after treatment with ASC or ASC^miR-210^ 7 days after I/R injury and subsequent sacrifice 2 weeks later, we observed improved myocardial function. However, the fibrotic area ratio to the left ventricle showed no significant changes (Supplementary Fig. 3). We believe that the miR-210 secreted from ASCs to EVs at the beginning of I/R helps to improve myocardial regeneration and function.

### MiR-210 derived from ASCs alters cell death via PTP1B and DAPK1

**Fig. 3:**
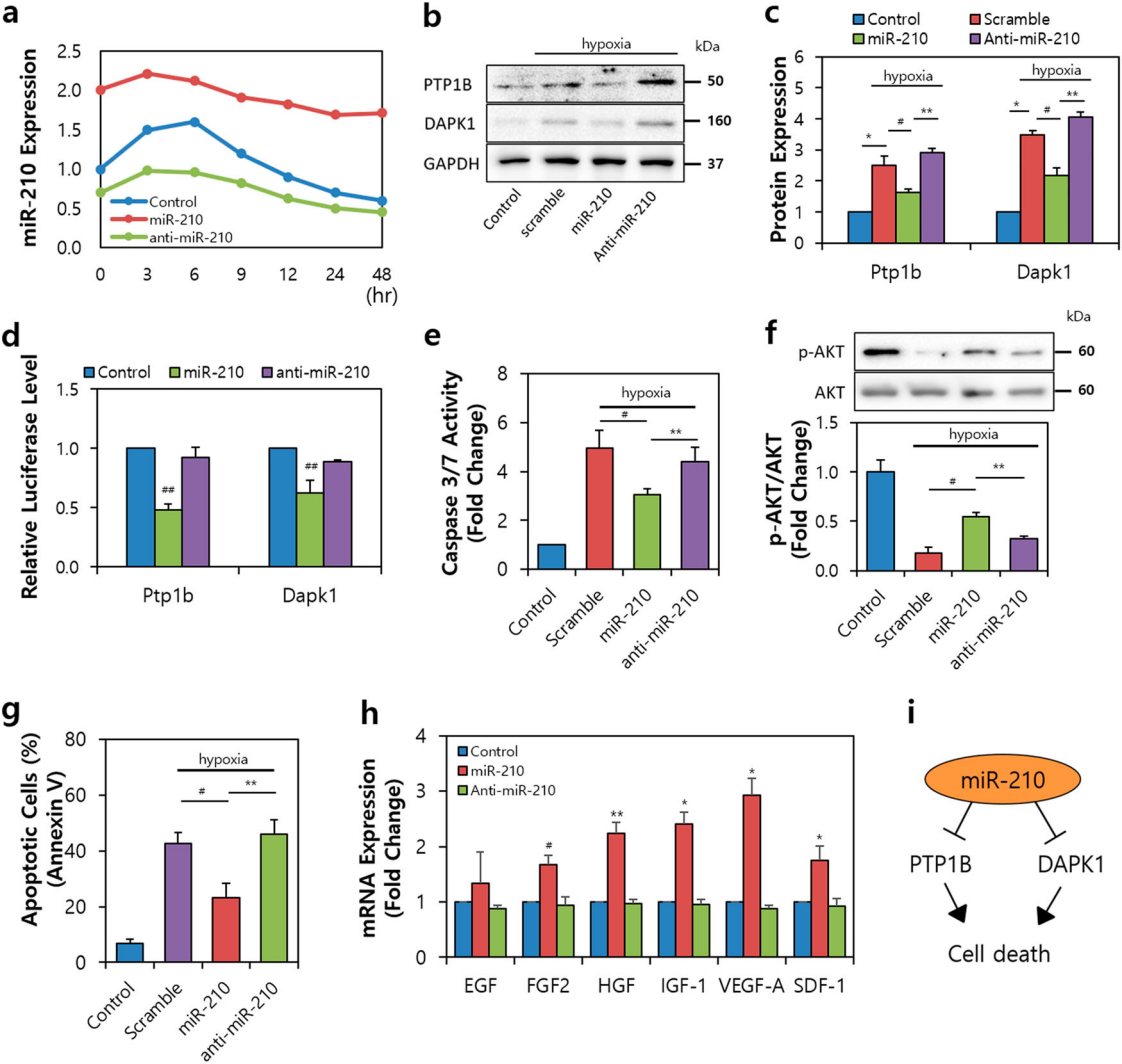
MiR-210 derived from ASCs regulates cell death via PTP1B and DAPK1. **a** Expression of miR-210 in ASCs treated with miR-210 or anti-miR-210 for 48 h. **b** Change in cell death-related proteins in ASCs under hypoxic conditions. **c** Protein expression of PTP1B and DAPK1 in ASCs after miR-210 or anti-miR-210 treatment. **d** Luciferase activity in the miR-210 or anti-miR-210 group. **e** Caspase 3/7 activity after miR-210 or anti-miR-210 treatment under hypoxia. **f** Expression and phosphorylation of Akt in hypoxic ASCs. **g** Population of apoptotic cells labeled with Annexin V. **h** Gene expression of growth factors and cytokines (EGF, FGF2, HGF, IGF-1, VEGF-A, and SDF-1) in transfected ASCs. **i** Summary of anti-cell death signaling regulated by miR-210 in ASCs. PTP1B protein tyrosine phosphatase 1B, DAPK1 death associated protein kinase 1, EGF epidermal growth factor, FGF2 fibroblast growth factor, HGF hepatocyte growth factor, IGF-1 insulin-like growth factor 1, VEGF-A vascular endothelial growth factor A, SDF-1 stromal cell-derived factor 1. *P < 0.05 control vs. scramble, ^#^*P* < 0.05 scramble vs. miR-210, **P < 0.05 miR-210 vs. anti-miR-210, ^##^*P* < 0.05 vs. control. All values are the mean ± s.d. Statistical significance was assessed by one-way ANOVA.

To investigate the effect of miR-210 in ASCs, we induced hypoxic conditions in vitro when ASCs were transplanted into the I/R-injured hearts. Our results revealed altered expression levels of miR-210 in ASCs after transfection with miR-210 or anti-miR-210 (Fig. 3a). In the miR-210-transfected group, the rate of miR-210 expression remained stable until 48 h of hypoxic treatment, whereas the rate of miR-210 rapidly decreased after 6 h of hypoxic treatment in the control group; furthermore, miR-210 was continuously expressed at lower levels than normal in the anti-miR-210-transfected group.

The PTP1B and DAPK1 genes are known representative targets of miR-210^24^. To demonstrate the mechanism of miR-210 in ASCs, we reconfirmed the target genes for miR-210 using both the TargetScan and miRWalk algorithms. Next, we investigated these factors using biochemical assays based on immunoblots (Fig. 3b, c) and luciferase activity (Fig. 3d). Our results revealed that under hypoxic conditions, PTP1B and DAPK1 showed decreased expression in the miR-210 group compared to the anti-miR-210 groups. Luciferase activities were significantly decreased by miR-210 in all cases, indicating that both proteins were direct targets of miR-210. In addition, miR-210 exposure resulted in decreased caspase 3/7 activity in the ASCs transfected with miR-210 compared with scramble and anti-miR-210 under hypoxia (Fig. 3e). Conversely, the rate of Akt phosphorylation indicating cell survival was increased in the miR-210 group (Fig. 3f) compared with the scramble and anti-miR-210 groups. Fluorescence-activated cell sorting analysis also showed fewer apoptotic cells (Fig. 3g) than those in the scramble and anti-miR-210 groups.

Since miR-210 is known to be associated with angiogenesis-associated factors, we focused on the miR-210 function in ASCs^24,25^. Using real-time PCR analysis, we observed that miR-210 expressed angiogenic factors but not EGF (Fig. 3h). The mRNA expression ratio was increased compared to that of the control group in the following ascending order: FGF2, 1.72-fold; HGF, 2.31-fold; IGF-1, 2.49-fold; VEGF-A, 2.94-fold; and SDF-1, 1.81-fold. Taken together, these data demonstrated that miR-210 modulates apoptotic inhibition and angiogenic promotion via PTP1B and/or DAPK1 (Fig. 3i).

### MiR-210 in EVs from ASCs participates in the angiogenic promotion of HUVECs

**Fig. 4:**
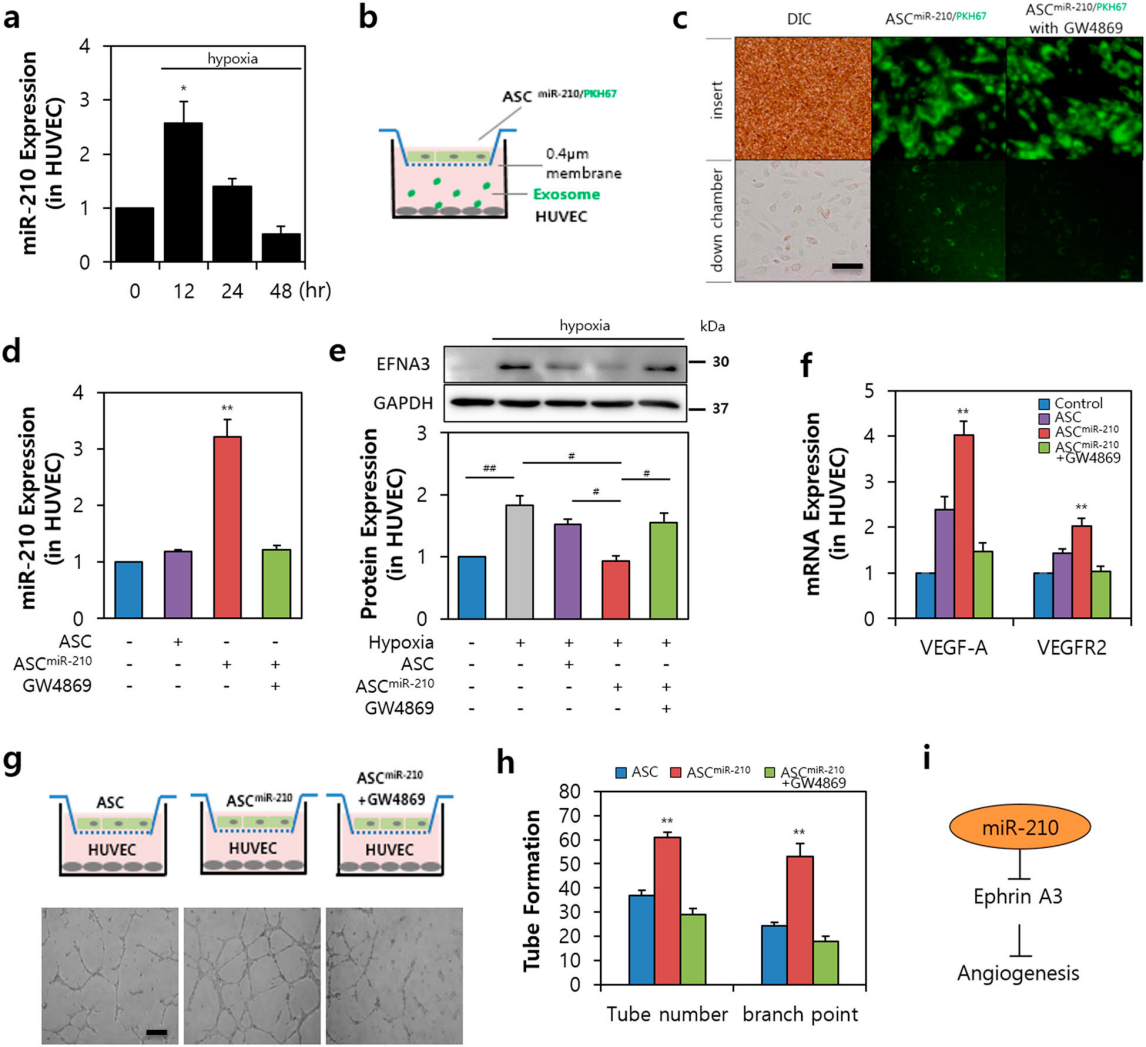
EV-210 contributes to angiogenesis by inhibiting Ephrin A3 in damaged endothelial cells. **a** Expression of miR-210 during 48 h of incubation under hypoxic conditions. **b** Exosome permeable system for ASC^miR-210/PKH67^ cocultured with HUVECs. MiR-210 was labeled with PKH67 dye (green) in ASCs. **c** EV-210 (upper panel, insert) permeabilization to HUVECs (lower panel, lower chamber). Left lane: differential interference contrast (DIC); middle and right: green wavelength of confocal microscopy. Bar = 100 μm. **d** Expression of miR-210 in HUVECs after transfection of miR-210 and/or GW4869 into ASCs. **e** Change in EFNA3 expression in hypoxic HUVEC. **f** Change in angiogenic gene expression, VEGF-A (left) and VEGFR2 (right) in HUVECs. **g** Capillary tube formation from HUVECs using an exosome permeable system. Bar = 100 μm. **h** Results of tube number and branch point in HUVECs. i Summary of angiogenesis regulated by ASC-miR-210 (EV-210) via Ephrin A3 in HUVECs. VEGF-A vascular endothelial growth factor A, VEGFR2 vascular endothelial growth factor receptor 2, EFNA3 Ephrin A3. *P < 0.05 vs. 0 h, **P < 0.05 vs. the control, ^#^*P* < 0.05, ^##^*P* < 0.001. All values are the mean ± s.d. Statistical significance was assessed by one-way ANOVA.

First, we investigated the angiogenic effect in vitro by simulating damaged endothelial cells. We observed that miR-210 expression peaked in HUVECs at up to 12 h of hypoxia, after which it decreased in a time-dependent manner (Fig. 4a). To confirm the effect of miR-210 isolated from the EVs of ASCs, we exposed HUVECs to EV-producing conditions by applying an indirect method (Fig. 4b)^22^. The miR-210-transfected ASCs were stained with PKH67 dye, attached to a 0.4 µM membrane, and incubated in a lower chamber of HUVECs to examine the effect of EVs from the insert (Fig. 4c). We observed that EVs from ASC^miR-210/PKH67^ had diffused to the lower chamber containing HUVECs; however, no green fluorescence was found in the group subjected to GW4869 treatment. HUVECs located in the lower chamber of ASC^miR-210/PKH67^ had a 3.31-fold greater expression of miR-210 than that of the ASC-only group (Fig. 4d).

Ephrin A3 (Efna3) production is a known consequence of miR-210 modulating the endothelial cell response^26^. The ASC^miR-210^ group further showed a similar Efna3 expression ratio to that of the normoxic group. However, ASC^miR-210^ treated with GW4869 showed no secretion of EV, and consequently, the expression ratio of Efna3 recovered to the level of the ASC group (Fig. 4e). Furthermore, angiogenic factors (Vegf-a and Vegfr2) were upregulated in the ASC^miR-210^ group compared to the normoxic group (Fig. 4f; 4.03-fold and 2.18-fold, respectively). To examine the effect of ASC^miR-210^ on angiogenesis, we incubated HUVECs in chamber culture dishes to perform a tube formation assay (Fig. 4g). The results showed that the tube number and branch point in the ASC^miR-210^ group were significantly increased compared to those in the ASC group (Fig. 4h). Thus, our results indicate that the proangiogenic activity of EVs from ASC^miR-210^ is mediated by miR-210 activity via Ephrin A3 (Efna3; Fig. 4i).

### MiR-210 in EVs from ASCs prevents cell death of H9c2 cells under hypoxia

**Fig. 5:**
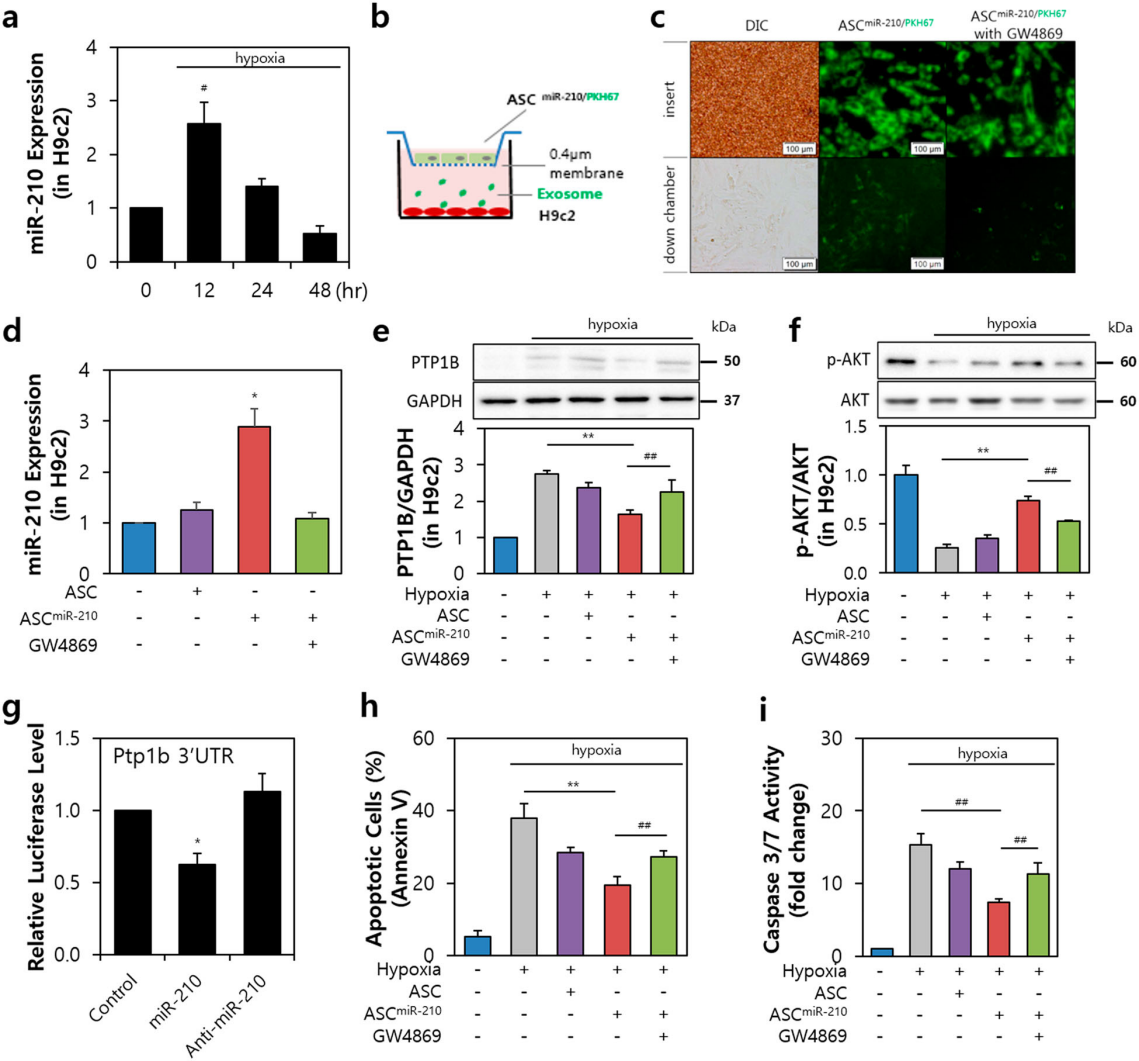
EV-210 increases cardiac muscle survival via PTP1B. **a** Expression of miR-210 after 48 h of incubation under hypoxic conditions in H9c2 cells. **b** Exosome permeable system for ASC^miR-210/PKH67^ cocultured with H9c2 cells. MiR-210 was labeled with PKH67 dye (green) in ASCs. **c** EV-210 (upper panel, insert) permeabilization to H9c2 cells (lower panel, lower chamber). Bar = 100 μm. **d** Expression of miR-210 in H9c2 cells after transfection of miR-210 or treatment with GW4869 into ASCs. **e** Change in PTP1B expression in hypoxic H9c2. **f** Expression and phosphorylation of Akt in hypoxic H9c2 cells. **g** Luciferase reporter assays indicated that miR-210 regulates Ptp1b expression by targeting the estimated target sites. **h** Population of apoptotic cells labeled with Annexin V in H9c2 cells. **i** Caspase 3/7 activity in the ASC, ASC^miR-210^ or GW4869 groups. PTP1B, Protein tyrosine phosphatase 1B; 3′UTR, three prime untranslated region. ^#^*P* < 0.05 vs. 0 h, *P < 0.05 vs. the control, ^##^*P* < 0.05, **P < 0.001^.^ All values are the mean ± s.d. Statistical significance was assessed by one-way ANOVA.

We found that miR-210 expression in H9c2 cells peaked at up to 12 h of hypoxia (2.53-fold vs. normoxia group) and subsequently decreased in a time-dependent manner (Fig. 5a). MiR-210 expression was confirmed using a chamber assay (Fig. 5b, c). We observed a significant increase in the expression level of the ASC^miR-210^ group compared to the ASC-treated or GW4869-treated groups (Fig. 5d; 2.91-fold vs. 1.31-fold or 1.17-fold, respectively).

PTP1B is involved in the induction of apoptosis^24,27^, and since ASC^mir-210^ regulates apoptotic signaling under hypoxic conditions, it was confirmed that the secreted EVs from ASC^miR-210^ also play a pivotal role. The expression ratio of PTP1B/GAPDH was decreased when compared to that in the hypoxia only group (57.09%) or hypoxic ASC ^miR-210^ treated with GW4869 group (71.88%) (Fig. 5e). Furthermore, the phosphorylation ratio of Akt was increased in the hypoxic ASC^miR-210^ group compared to the hypoxic ASCs (2.88-fold) or hypoxic ASC ^miR-210^ treated with GW4869 groups (1.24-fold) (Fig. 5f). Furthermore, exposure to miR-210 significantly reduced the luciferase activities of wild-type PTP1B by 35.55% compared with the control (miR-scramble) (Fig. 5g). To examine the apoptotic changes induced by EVs from ASC ^miR-210^, we analyzed the ratio of apoptotic cells and the activity of caspase 3/7. In the hypoxic ASC ^miR-210^ group, the ratio of apoptotic cells was decreased compared to that in the hypoxia only group or hypoxic ASC ^miR-210^ treated with GW4869 group (Fig. 5h). Additionally, the activity of caspase 3/7 was attenuated in the hypoxic ASC ^miR-210^ group compared to the hypoxia only group (0.48-fold) or hypoxic ASC ^miR-210^ treated with GW4869 group (0.62-fold) (Fig. 5i). We therefore conclude that EVs from miR-210-transfected ASCs control the survival and antiapoptotic effect of cardiac muscle cells via PTP1B.

### MiR-210 in EVs from ASCs selectively acts on neighboring cardiac fibroblasts

Subsequent to MI, increased collagen deposition at the site of tissue damage by TGF-β activation causes diastolic stiffness^28^. We found that TGF-β expression and cell proliferation increased approximately 1.5-fold and 1.62-fold, respectively, in the I/R-injured hearts (Supplementary Fig. 4a, b). In addition, it has been reported that the profibrotic effects of TGF-β can be mediated through an increase in connective tissue growth factor (CTGF)^29^. Our studies revealed that TGF-β stimulation of cardiac fibroblasts improved the mRNA expression of CTGF (1.73-fold), and the expression ratio of CTGF/GAPDH (1.41-fold) (Supplementary Fig. 4c, d).

**Fig. 6:**
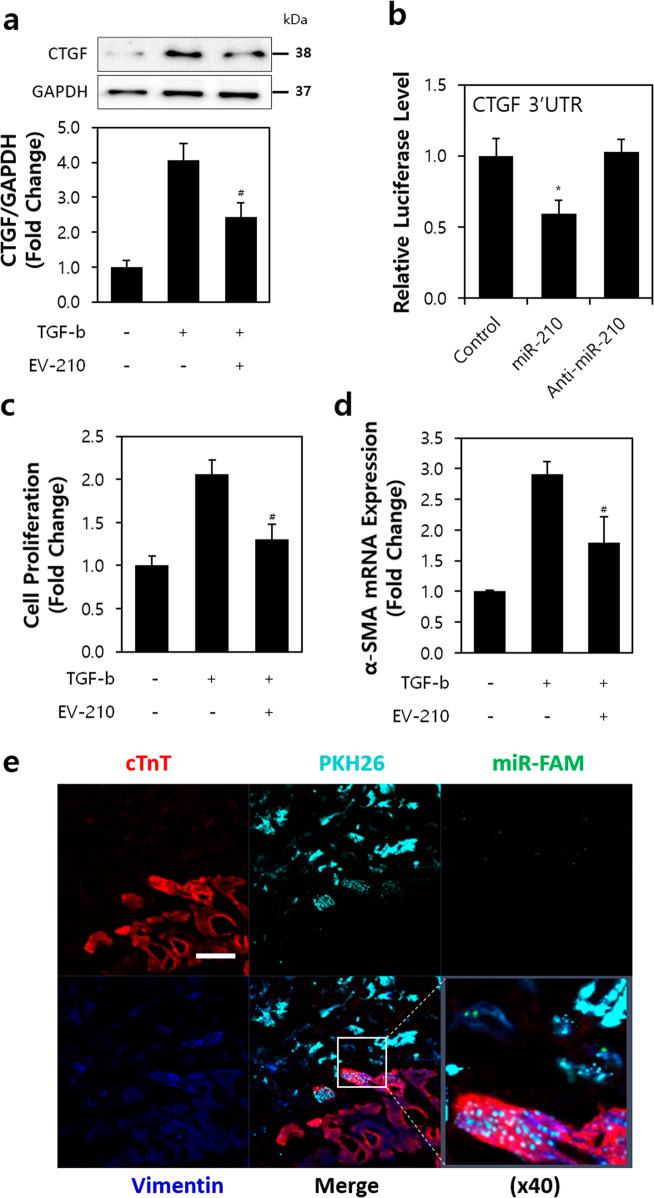
EV-210 has a selective antiproliferative effect on cardiac fibroblasts. **a** Expression of CTGF in cardiac fibroblast. **b** Relative luciferase level of the CTGF 3’UTR after treatment with miR-210 or anti-miR-210. **c** Change in fibroblast proliferation with TGF-b and/or EV-210. **d** Expression of α-SMA mRNA levels in cardiac fibroblasts. **e** Internalization of EV-210 (miR-FAM, green) onto fibroblasts (vimentin, blue). ASC, light blue; cTnT, red. Magnification = ×10 and ×40. Bar = 50 μm. CTGF connective tissue growth factor, TGF-b transforming growth factor beta, α-SMA α-smooth muscle actin, cTnT cardiac troponin T, PKH26, yellow-orange fluorescent dye with long aliphatic tails reagent. ^#^*P* < 0.05 vs. the TGF-b- and EV-210-negative group, *P < 0.05 vs. the control. All values are the mean ± s.d. Statistical significance was assessed by one-way ANOVA.

Post-treatment with EV-210 under TGF-β stimulation resulted in a reduction in CTGF/GAPDH expression by 60% (Fig. 6a). The luciferase activity significantly decreased by approximately 42% after miR-210 exposure compared to that of the control group, indicating that CTGF is a direct target of miR-210 (Fig. 6b). Cell proliferation increased approximately 0.62-fold (EV-210-treated group) compared with that of the untreated group (TGF-β only) (Fig. 6c). In the proliferative phase of fibrosis, α-smooth muscle actin (α-SMA) expression is markedly increased in the fibrotic areas^30^. Our data revealed that the expression ratio of α-SMA was significantly reduced in association with the reduction in cardiac fibroblast proliferation by EV-210 (0.62-fold; vs. TGF-β only group) (Fig. 6d).

Using 6-FAM fluorescein-conjugated miR-210 (miR-FAM), we transfected ASCs and then stained them with PKH26. After 1 h of I/R, rats were treated with ASC^miR-210^ and sacrificed to obtain the stem cell injection site after 3 weeks. Examination of the secretions from the injection site identified miR-210 (FAM, green) in EVs derived from ASC^miR-210^ (PKH26, light blue) from cardiac muscle cells (cTnT, red) and cardiac fibroblasts (Vimentin, blue) in the I/R-injured heart (Fig. 6e). Vimentin, an intermediate filament of fibroblasts, is the most widely used fibroblast marker^31^. An overlap of fibrotic cells with cardiomyocytes was observed in the lower part, and ASCs stained with PKH26 were distributed in the upper region. MiR-FAM derived from EVs of ASC^miR-210^ (green) was identified at the border but not in cardiac fibroblasts (lower region). Furthermore, a red dot, which is EVs at the interface between PKH26-labeled ASC^miR-210^ (red) and fibroblasts (green, vimentin), appeared on top of the cytoplasm in ASCs but was absent on the cell surface of fibroblasts (Supplementary Fig. 4e). The expression of miR-210 in cells after treatment with ASC-conditioned or ASC^miR-210^-conditioned medium was 3.7 and 8.2 times higher in H9c2 cells and HUVECs, respectively but was not significantly different in cardiac fibroblasts (Supplementary Fig. 4f). These results suggest that EVs derived from miR-210-transfected ASCs have selective intercommunication with myocardial fibroblasts, but not cardiac muscle and endothelial cells, but this appears to be a trophic effect through cell secretion rather than a direct effect of EVs through cell interaction.

### Therapeutic silencing and extracellular transfer of miR-210 ameliorates LV dysfunction after I/R injury

**Fig. 7:**
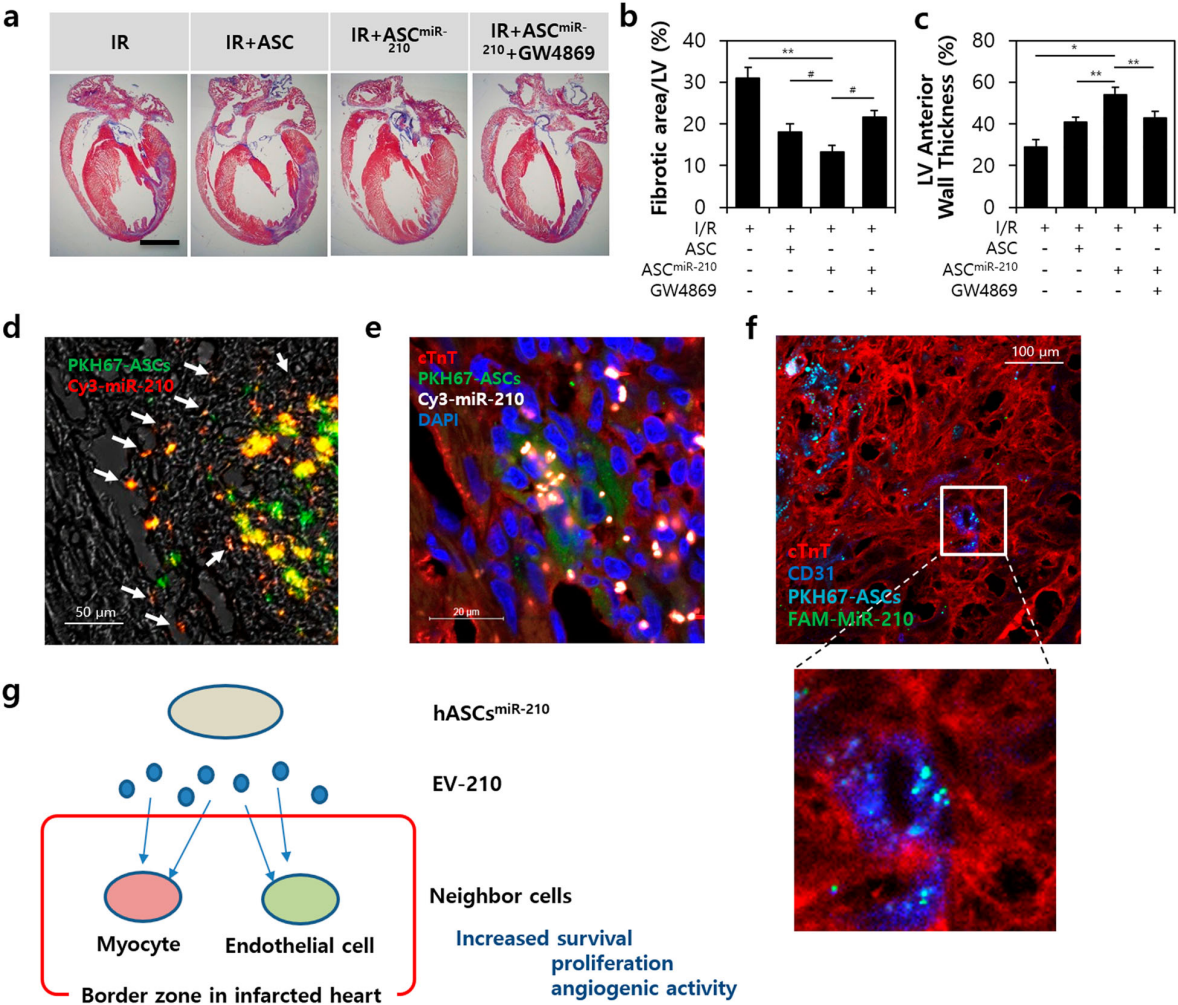
EV-210 attenuates LV remodeling and activates cardiac regeneration in ischemia-reperfusion hearts. **a** Trichrome-stained sections after 3 weeks of IR and transplantation of ASCs. Bar = 3 mm. **b** Fibrotic area of left ventricles. **c** Change in left ventricle anterior wall thickness. **d** Colocalization between ASC (PKH67, green) and miR-210 (Cy3, red). Bar = 50 μm. White arrow, EV-210 (orange). **e** Internalization of cardiac muscle cells (cTnT, red) and ASCs (PKH67, green). EV-210 (cy3, yellow). Bar = 20 μm. DAPI, blue. **f** Internalization of ASCs (PKH67, light blue) and EV-210 (FAM-miR-210, green) between cardiac muscle (CTnT, red) and small vessels (CD31, blue). Magnification = ×40. Bar = 100 μm. **g** Summary of cardiac regeneration in the border zone of infarcted hearts by exosomes with stem cell-derived miR-210. *P < .05, **P < .001, ^#^*P* < .005. All values are the mean ± s.d. Statistical significance was assessed by one-way ANOVA.

We confirmed the function of ASCs with miR-210 in the rats hearts with I/R injury (Fig. 7a). After ASC^miR-210^ treatment, we analyzed the fibrotic area/LV and LV anterior wall thickness. In the ASC^miR-210^ group, a significant decrease was observed in the fibrotic area (13.24 ± 2.41%) compared to that of the I/R only (31.97 ± 4.41%), ASC treatment (17.87 ± 3.01%), or ASC^miR-210^ with GW4869 groups (22.14 ± 2.21%) (Fig. 7b). Furthermore, the LV anterior wall thickness was dramatically increased (55.44 ± 2.30%) compared to that of the I/R only (28.31 ± 2.41%), ASC treatment (39.66 ± 1.68%), or ASC^miR-210^ with GW4869 groups (42.18 ± 1.87%) (Fig. 7c).

To identify miR-210 in EVs secreted in ASC^miR-210^, we applied PKH67 dye (green) to stem cells, and Cy-3 fluorescence (red) was applied to miR-210. The orange spot (white arrow) where the green and red are merged is estimated to be the miR-210 derived from the EVs of ASCs around the ischemic myocardium (Fig. 7d). To identify the effect of miR-210 on cardiac muscle cells, we performed fluorescent staining using cTnT (red), ASCs (PKH67), Cy3-miR-210 (white), and DAPI (blue) (Fig. 7e). MiR-210 (white spot) was abundantly found in ASCs and at the border between stem cells and cardiac muscle cells. Furthermore, miR-210 from ASCs between vessels in ischemic myocardium was identified by staining with cTnT (red), CD31 (blue), PKH67 (light blue), and FAM-miR-210 (green) (Fig. 7f). A number of EVs (aqua blue spot) containing miR-210 (PKH67 merged with FAM-miR-210) were found in the capillaries.

A unique function of EVs is to mainly mediate cell-to-cell communication (EV adhesion molecule)^13,14,32^. B cell-derived exosomes express β1 and β2 integrins, which are capable of mediating anchorage to the extracellular matrix^33^. Furthermore, integrin α4 contributes to the activation of exosome-induced endothelial cells^34^. Thus, we examined the binding between cell-to-cell and cell-EV interactions, which induce or change cell signaling (Supplementary Fig. 5). EVs were shown to express the α4 protein and β2 mRNA of integrin. VCAM-1 was highly expressed in HUVECs compared to H9C2 cells and fibroblasts, and ICAM-1 was evenly expressed in all cardiac cells except HUVECs. These results illustrate that miR-210 in EVs derived from ASC^miR-210^ has an important role in communication between neighboring cardiac cells at the border zone in infarcted hearts, thereby increasing survival, proliferation, and angiogenic activity (Fig. 7g).

## Discussion

Hypoxia is a common characteristic of several regenerative organisms and is required for maintaining the proliferative capacity of various cell types, including cardiomyocytes and endothelial cells, and improving numerous trophic factors^16,35^. Hypoxic preconditioning in stem cells has been reported to increase survival and angiogenesis^36^. Appropriate mitochondrial-derived reactive oxygen species (ROS) protect cardiovascular cells from oxidative DNA damage^35,37^. Recently, several groups identified miRs as crucial gene regulators in response to ischemic conditions by ROS^16,38^. Our studies identified specific miRs that induce survival and angiogenic functions (Fig. 1 and Supplementary Fig. 1a). Finally, we screened two candidate miRs with low expression rates in early hypoxic conditions that are rarely expressed in EVs of ASCs based on our standards. Previous studies have reported that miR-210 induces proliferation, survival, and angiogenesis in MI, and miR-210 uptake of bone marrow mesenchymal stem cells (MSCs) by preconditioning sevoflurane has also been reported^39^. However, proper delivery of miR-210 from ASCs and its effect on each cardiovascular cell remain unknown. The second candidate considered, miR-21, was later excluded since it was reported to cause myocardial hypertrophy when expression levels increased in cardiomyocytes^40^.

The paracrine effect of MSCs was first identified by Haynesworth et al.^41^. These researchers showed that MSCs produce and release various growth factors, chemokines, and cytokines that specifically signal adjacent cells. MSCs directly or indirectly induce paracrine signaling to reconstruct wounded tissues and to accelerate positive signals. The current study shows that the molecules secreted by MSCs act as mediators to activate target cells directly or stimulate neighboring cells indirectly to secrete active factors^42^. However, MSCs have been found to deliver information to damaged cells, to participate in tissue regeneration and to emit numerous EVs with biological activity similar to MSCs. Studies using EVs derived from MSCs commenced with the introduction of a model of I/R injury of the heart in 2010^43^. Furthermore, stem cells have evolved a unique paracrine mechanism that recapitulates specific functions by transporting miRs through EVs. This process includes regulatory effects derived from MSC-derived EVs with miR, including differentiation, self-renewal, pluripotency, maturation, and cell fate determination. Cargo-enriched MSC exosomes were used to carry out the virus-free miR-199 loading approach, inducing proliferation and reducing death in cardiomyocytes^44^. Furthermore, it has been reported that miR-22-loaded exosomes are transplanted into MSCs to inhibit cardiac fibrosis through direct targeting of methyl CpG binding protein 2^45^. In endothelial cells, the exosomal transferred miR-125a of MSCs represses DLL4 expression and modulates angiogenic functions by promoting the formation of endothelial tip cells^46^. These studies suggest that “one” type of miR affects “one” type of cardiovascular cell through “one” signaling pathway.

MiR and EV share some common characteristics. Since EVs act in combination with various types of proteins and RNAs, their responses are more complex and longer lasting than those induced by individual molecules. Because individual miRs target multiple mRNAs in a similar manner, manipulation of miRs can have a significant impact on intracellular networks. In addition, EVs act as an extracellular reservoir that releases specific molecules with different spatial and temporal distribution profiles compared to whole cells. The miR-dependent regulation of cell signaling acts like an ID card that directs the production of trophic factors, such as proteins and growth factors to improve various diseases^47,48^. We named the common role of miRs and EVs a “multiplexed target”. In this study, “one” miR of EVs derived from ASCs was selected and analyzed for “various” cell signals that respond positively to “various” cells.

This study investigated the forward function of miR-210 in ASCs, which serves to produce and deliver functional EVs. PTP1B biologically links PTP and its substrates and may provide opportunities for the development of new therapies for human disease; this molecule may also be important in pathological processes such as cell proliferation and survival^27,49^. DAPK1 belongs to a family of calcium/calmodulin (Ca^2+^/CaM)-regulated Ser/Thr kinases and is considered a multidomain protein. In addition to the kinase and CaM regulatory domains, DAPK1 has been reported to include a C-terminal death domain capable of regulating cell death, survival, and proliferation. As suggested above, we found that ASC^miR-210^ modulates PTP1B or DAPK1 to decrease cell death (Fig. 3). In endothelial cells, we examined the process and effects of secreted EVs from ASC^miR-210^ on recipient cells. Ephrin ligand and its receptor were observed to play a crucial role in cardiovascular development and angiogenesis^50^. Furthermore, previous studies have shown that miR-210-dependent downregulation of EFNA3 expression contributes to the regulation of angiogenic responses to ischemia^51^. We found that ASC^miR-210^ modulates a crucial element of the endothelial cell response to hypoxia, which affects the angiogenic effect via EFNA3 (Fig. 4). Furthermore, we observed that ASC^miR-210^ regulates cell survival and the antiapoptotic response through changes in PTP1B in cardiac muscle cells (Fig. 5). The process and effects of secreted EVs from ASC^miR-210^ on recipient cells were also examined in cardiac fibroblasts. CTGF has been shown to induce the proliferation of cardiac fibroblasts and extracellular matrix production in connective tissue^52^. In acute MI, CTGF expression increases proportionately to collagen^53^. Our study revealed that ASC^miR-210^ reduces CTGF expression and cell proliferation via miR-210 activation (Fig. 6a–d); however, EVs derived from ASC^miR-210^ do not appear to be docked in cardiac fibroblasts (Fig. 6e), suggesting that it is a paracrine effect induced by ASC^miR-210^.

The finding that EVs contain proteins, messenger RNAs and miRs suggests a mediating role in intercellular communication. EVs are able to move between different cells and affect the physiological pathways of recipient cells using specific information from donor cells^14,54^. A recent study found that overexpression of integrin α4β1 on primary melanoma cells is associated with increased bone turnover through interaction with constitutively expressed VCAM-1 in bone marrow stromal cells^55^. We found a similar coexpression of ITGA4 and VCAM-1 in HUVECs. The β2/α heterodimer interacts with the opposite receptors of adjacent cells, namely, ICAM-1, to regulate cell–cell adhesion or bind to ECM proteins. Furthermore, it has been reported that the β2 integrin subfamily may have other activities in addition to leukocyte attachment, such as regulating cell migration and differentiation during heart valve and heart myocardial wall morphogenesis. In our study, we found coexpression of LFA-1 and ICAM-1 in cardiac muscle cells (Supplementary Fig. 5). Although further studies are required, we were able to indirectly examine intercellular communication between EVs and cardiovascular cells.

In summary, our study reveals that miR-210 derived from EVs of ASC^miR-210^ improves heart function by inhibiting apoptosis and upregulating angiogenesis as well as the trophic effect of ASC itself. EVs and miRNAs are multiplexed targeting tools that are accurate and selective, and temporal delivery can affect an entire gene network and modify the complex status of various cell types. Our approach shows that multiplexed targeting of miR-210 through EVs derived from stem cells has the potential to be a novel therapy for ischemic heart diseases.

## Supplementary information

Supplementary Information
