# Supplementary material for: Multiplexed targeting of miRNA-210 in stem cell-derived extracellular vesicles promotes selective regeneration in ischemic hearts
Source: Exp Mol Med. 2021 Apr 20;53(4):695–708. doi: 10.1038/s12276-021-00584-0 (PMC8102609; doi:10.1038/s12276-021-00584-0)
Supplement: Supplementary file 1 — Supplementary Information [file 12276_2021_584_MOESM1_ESM.docx]

**- Supplementary Information -**

**Multiplexed targeting of miRNA-210 in stem cell-derived extracellular vesicles promotes a selective regeneration in ischemic heart**

Byeong-Wook Song^1^, Chang Youn Lee^2^, Ran Kim^3^, Min Young Lee^4^, Jongmin Kim^5^,

Jee-Yeong Jeong^6^, Woochul Chang^3^

^1^Institute for Bio-Medical Convergence, Catholic Kwandong University International St. Mary’s Hospital, Incheon, Republic of Korea

^2^Department of Integrated Omics for Biomedical Sciences, Graduate School, Yonsei University, Seoul, Republic of Korea

^3^Department of Biology Education, College of Education, Pusan National University, Busan, Republic of Korea

^4^Department of Molecular Physiology, College of Pharmacy, Kyungpook National University, Daegu, Republic of Korea

^5^Department of Life Systems, Sookmyung Women’s University, Seoul, Republic of Korea

^6^Department of Biochemistry, Kosin University College of Medicine, Busan, Republic of Korea

**Correspondance :** Woochul Chang ([wchang1975@pusan.ac.kr](mailto:wchang1975@pusan.ac.kr))

**Supplementary materials**

**Stable overexpression of miR-210 in hASCs**

hASCs were stably transduced with commercially available shMIMIC lentiviral microRNA particles expressing miR-210 (has-miR-210 shMIMIC), termed as miR-210 shMIMIC lentiviral particles, termed as EV MIMIC (Thermo Fisher Scientific, Waltham, MA). The plasmid is based on the pSMART vector with a puromycin selection and GFP cassette. Cells were transduced using OptiMEM medium (Life Technologies, NY) which contained 6 μg/ml polybrene (Sigma-Aldrich, St. Louis, MO) with lentiviral particles at multiplicity of infection (MOI) = 8. On day 4 post-transduction, puromycin (Sigma-Aldrich) selection was initiated and GFP-positive clones were selected using cloning cylinders. Cells were plated in 24-well plates for expansion and propagated at a final concentration of 200 μg/ml puromycin.

**MicroRNA microarray assay**

Total RNA from hASC-derived EVs was extracted using miRNeasy mini kit (Qiagen, Valencia, CA) according to the manufacturer’s instructions. The quantity and quality of RNA was determined by measuring using NanoDrop Lite (Thermo Fisher Scientific). The purified RNA were sent to System Biosciences and the miR custom array profiling services were provided.

**Immunoblot analysis**

The cells were washed in PBS and then lysed in RIPA lysis buffer (Thermo Fisher Scientific), 1% phosphatase inhibitors and 1% protease inhibitors. Protein concentrations were determined using the BCA Protein Assay kit (Thermo Fisher Scientific). Briefly, 20 µg protein was subjected to 8% or 10% sodium dodecyl sulfate-polyacrylamide gel electrophoresis (SDS-PAGE) and then transferred to a polyvinylidene difluoride membrane (Millipore). The membrane was blocked with Tris-buffered saline/Tween 20 (TBS-T, 0.05% Tween 20) and 5% skim milk for 1 hr at room temperature and then incubated with the appropriate primary antibodies overnight at 4°C. The following antibodies were used in these experiments: anti-PTP1B (SC-133259) and anti-CTGF (SC-365970) from Santa Cruz Biotechnology (Santa Cruz, CA). The membrane was washed three times with 0.01% TBS-T for 5 minutes and then incubated with skim milk and horseradish peroxidase-conjugated secondary antibodies (Santa Cruz Biotechnology) for 1 hr at RT. After the membrane was washed six times for 5 minutes each, the bands were detected with an enhanced chemiluminescence reagent (ECL; GE healthcare, Chicago, IL). The band intensities were quantified using a photo-image system (Molecular Dynamics) and NIH ImageJ version 1.48v software.

**RNA isolation and reverse transcription PCR**

Total RNA was isolated from ASCs, cardiomyocytes and HUVECs using TRIzol reagent (Ambion, Waltham, MA) according to the manufacturer’s instructions. The concentration of RNA was determined by measuring using NanoDrop Lite (Thermo Fisher Scientific). Oligo dT-primed cDNA was synthesized from total RNA using the Maxime RT PreMix kit (iNtRON Biotechnology, Seongnam, Korea). The cDNA synthesis and RTase inactivation were performed for 1 h at 45°C and for 5 min at 95°C, respectively.

**Quantitative real-time PCR (qPCR)**

Amplification and detection of specific products were performed on a StepOnePlus Real-time PCR System (Life Technologies) using *SYBR Premix Ex Taq II* (Takara Bio INC, Japan) at 95°C for 30 sec, followed by 40 cycles of 95°C for 5 sec and 60°C for 30 sec. The threshold cycle (Ct) of each target gene was automatically defined and normalized to control GAPDH (ΔCt value). The relative difference in expression levels of each mRNA in VSMCs (^ΔΔ^Ct) was calculated and presented as fold induction (2^−ΔΔ^Ct). Real-time PCR primers were listed as follows: EGF forward primer: 5’-GGT CTT GCT GTG GAC TGG AT-3’; EGF reverse primer: 5’-CTG CTA CAG CAA ATG GGT GA-3’; IGF-1 forward primer: 5’- TCA CCT TCA CCA GCT CTG C-3’; IGF-1 reverse primer: 5’-TGG TAG ATG GGG GCT GAT AC-3’; HGF forward primer: 5’-GCC TGA AAG ATA TCC CGA CA-3’; HGF reverse primer: 5’-GCC ATT CCC ACG ATA ACA AT-3’; FGF-2 forward primer: 5’-AGC GGC TGT ACT GCA AAA AC-3’; FGF reverse primer: 5’-CTT TCT GCC CAG GTC CTG TT-3’; VEGF-A forward primer: 5’-AGT CCA ACA TCA CCA TGC AG-3’; VEGF-A reverse primer: 5’-TTC CCT TTC CTC GAA CTG ATT T-3’; GAPDH forward primer: 5’-ACA TCG CTC AGA CAC CAT G-3’; GAPDH reverse primer: 5’-TGT AGT TGA GGT CAA TGA AGG G-3’.

**MicroRNA quantification**

In brief, 100 ng purified total RNA was used for reverse transcription (TaqMan® MicroRNA Reverse Transcriptase Kit, Applied Biosystems, Waltham, MA) in combination with TaqMan MicroRNA Assays to quantify miRNA-210 and U6 control transcripts according to the manufacturer's conditions. The threshold cycle (C_T_) of miR-210 and U6 expression was automatically defined, located in the linear amplification phase of the PCR, and normalized to the control U6 (ΔCt value). The relative difference in the expression level of miR-210 in the sorted cells (ΔΔC_T_) was calculated and presented as the fold induction (2 ^–ΔΔCT^).

**Luciferase assay**

The 3’ UTR sequence of PTP1B, CTGF, Efna3 and Dapk1 were amplified using each primers with Xho1 (forward) and Xba1 (reverse) endonuclease site. The each target gene 3’ UTR fragment was then cloned into the pMIR GLO vector. HeLa cells (ATCC, Rockville, MD) were plated in 24-well plates (Corning Incorporated, Corning, NY) at a density of 2×10^4^ cells per well. The pmirGLO vector the target binding site was co-transfected with miR-210 or the scrambled miRNA control (NC) using Lipofectamine 2000 (Invitrogen, Carlsbad, CA). Luciferase activity was measured 48 hrs later using a luminometer and the Dual Luciferase Assay (Promega Corporation, Madison, WI) according to the manufacturer's instructions. *Renilla* luciferase was used to normalize the cell number and transfection efficiency.

**EV transfer analysis by co-culture experiments**

ASCs (stained with PKH67) and H9c2 or HUVEC were co-cultured indirectly in two chambers separated by Falcon Cell Culture Inserts (BD Falcon, Franklin Lakes, NJ) to allow secreted EVs from ASCs to interact with in cardiomyocyte or HUVEC. Before co-culture, H9c2 or HUVEC were plates to the bottom chambers of 24-well plates at 2×10^4^ cells/well. H9c2 or HUVEC were pre-incubated in serum-free DMEM and EBM-2 medium, respectively. On the next day, hASCs were seeded to the top chambers at 1×10^4^ cells/insert. After co-culturing for 24 hours, they were observed under a fluorescence microscope (IX83, Olympus, Tokyo, Japan).

**Transfection of miRNA**

Transfection with miRNA-210 mimics or miRNA-210 inhibitors was performed using the TransIT-X2 system (Mirus Bio. LLC, Madison, WI). Mature miRNA-210 mimics (Genolution Pharmaceuticals, Korea) and miRNA-210 inhibitors (Integrated DNA Techologies, Coralville, IA) were used at final concentrations of 20 nM. After the cells were incubated for 24 hours, the media were replaced with fresh media for stabilization.

**Cell viability assay**

To measure cell viability, 5×10^3^ hASCs were plated in 96 well plate. The cells were transfected with 20nM miRNA mimics and incubated for 24 hrs. The plate was exposed to hypoxic conditions for 12 hours. Then, cell counting kit-8 reagent (CCK-8, Dojindo Molecular Technologies, Japan) was added to each well to a final concentration of 0.5 mg/mL, and the cells were incubated for 2 hours. The absorbance at 450 nm was measured using a microplate reader (Thermo Fisher Scientific).

**Annexin V/PI apoptosis assay**

To quantify apoptosis, 2×10^5^ hASCs were plated in 60 mm dishes and exposed to hypoxic conditions for 12 hrs. Then the cells were washed twice with PBS, and 1×10^5^ cells were resuspended in 100 μl of 1× binding buffer containing annexin V - FITC and propidium iodide (Apoptosis Detection kit, BD Biosciences) and then incubated for 15 min in the dark at RT. Then 2×10^4^ cells were analyzed by flow cytometry (BD ACCURI C6 cytometer, BD Biosciences). Annexin V-/PI-staining indicated the viable cells, annexin V+/PI- indicated the early apoptotic cells, and annexin V+/PI+ staining indicated the necrotic or late apoptotic cells.

**Measurement of caspase-3 activity**

Relative caspase-3 activity was determined by using caspase-3 activity assay kit (Roche, Switzerland). In brief, after different treatments, the cultured hASCs or H9c2 (5×10^5^) were extracted in 1 x DTT (USB Corporation, Cleveland, OH) for 1 min. Lysates were collected and loaded onto a plate coated with monoclonal caspase-3 antibody. Upon substrate cleavage, free fluorescent AFC (7-amino-4-trifluoromethylcoumarine) was quantified using a microplate reader (Bio-Rad).

**Tube formation**

A Matrigel-based tube formation assay was performed. Each well of a 96 well culture plate was coated with 50 μl of ECMatrix^TM^ (Millipore), which was allowed to incubate for 1 hr at 37°C. Then, HUVEC were seeded onto the coated wells at density of 1×10^4^ cells / well and cultured with 500 μl of EBM-2 with ASCs derived-EV or without EV and incubated at 37°C under 5 % CO_2_ for 12 hrs. After the incubation, tube formation images were captured by using a digital microscope camera system (Olympus, Japan).

**Immunofluorescence staining**

The heart tissues were fixed in 4% paraformaldehyde (PFA), overnight at 4°C. After washing with PBS for three times, the tissues were incubated with 30% sucrose overnight. Then tissues were embedded in OCT compound (Thermo Fisher Scientific) in cryomolds. Cryosections of 5 μm of thickness were obtained and used for immunofluorescence staining. The slides were allowed to stand at room temperature for 30 min and then fixed in cold acetone for 5 min. After air-drying for 30 min, rinse twice with PBS. After blocking with 2% horse serum solution for 20 minutes, slides were incubated with primary antibodies at 4°C for overnight. The antibodies that were used for the study include anti-cTnT(1:1000; Abcam, UK) and CD31 (1:2000; Sigma-Aldrich). All antibodies were diluted with 1 % BSA in PBS. Texas Red-conjugated goat anti-mouse IgG and rhodamine-conjugated goat anti-rabbit IgG (Jackson ImmunoResearch, West Grove, PA) were used as secondary antibodies. The slides were mounted with DAPI-contained mounting medium (Vector laboratory, Burlingame, CA). All images were made using an excitation filter under laser scanning confocal microscopy (LSM 700; Carl Zeiss, Germany) and transferred to a computer with Zen 2012 software (Carl Zeiss).

**Supplementary figures**

**
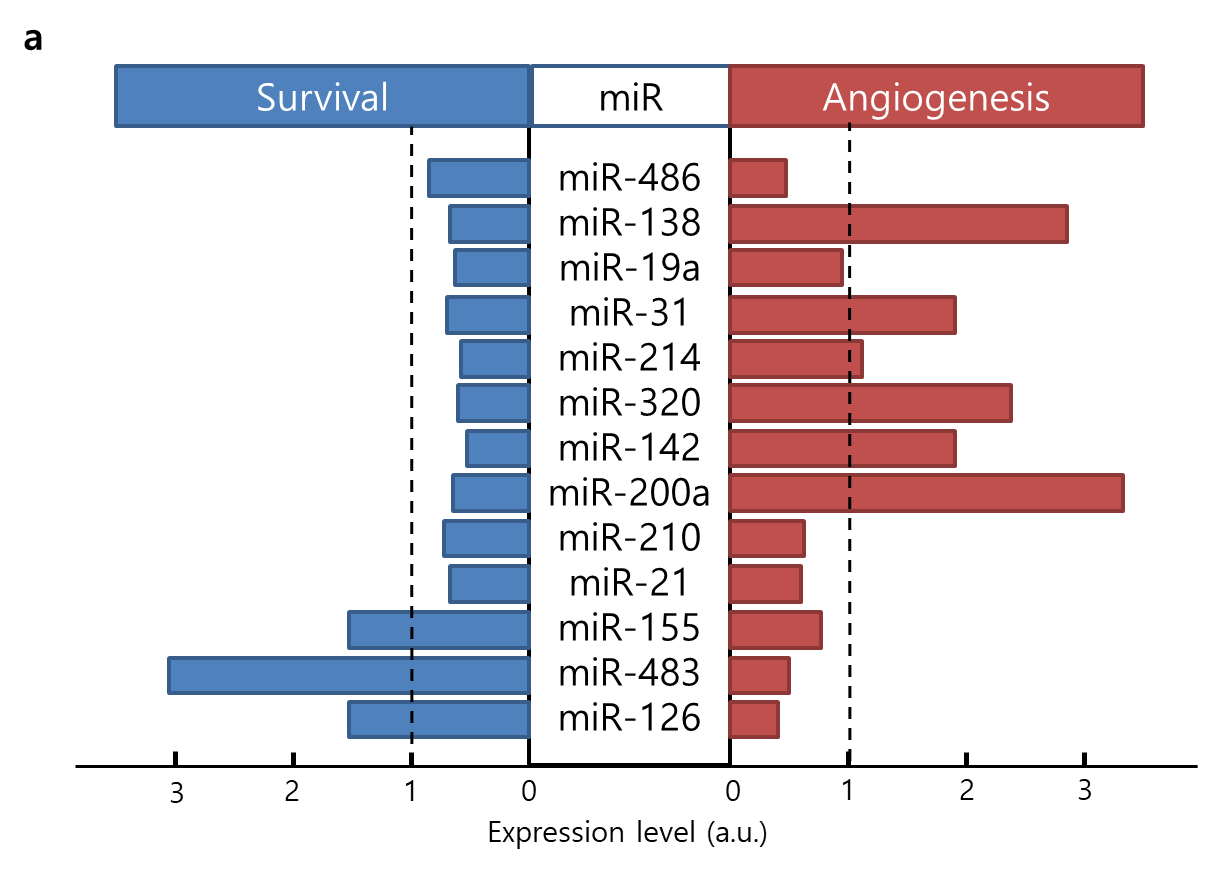
**

**
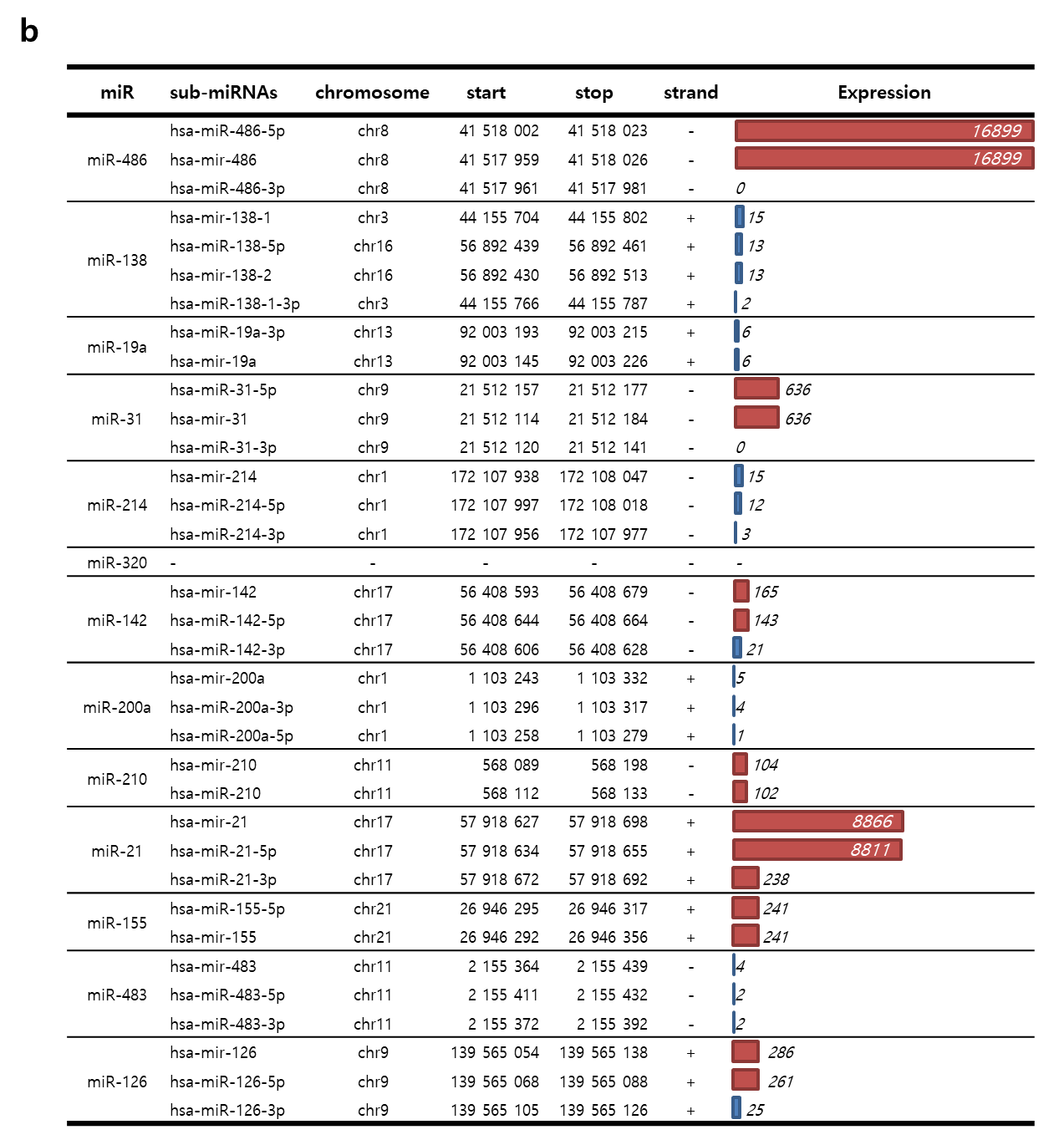
**

**Supplementary Fig. 1 MiR-210 and miR-21 is increased in the regulation of cardiac cells and EV derived from ASCs. a** Expression profile of miRNAs that regulated survival and angiogenic function in H9c2 under hypoxic condition. This analysis was confirmed by qPCR. **b** Validation of selected miRNAs that were distinctively changed in the miRNA array of EVs from normal ASCs. The chromosome number, strand and coordinates were taken from Genome Browser of the UCSC Genomic institute (<http://genome.scsc.edu>).

**
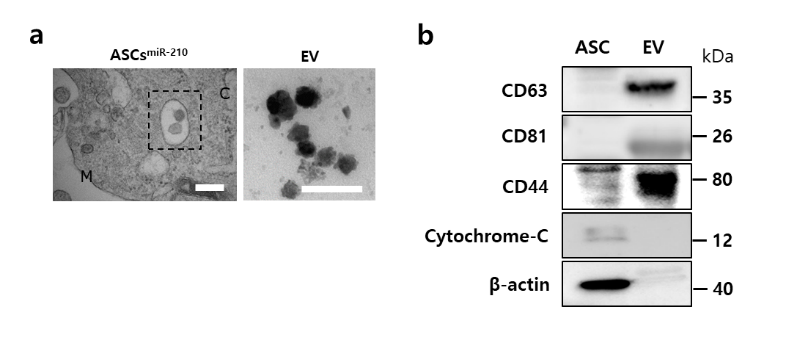
**

**Supplementary Fig. 2 MiR-210-transfected ASCs have normal EVs. a** Transmission electron microscopy (TEM) of EVs isolated from ASCs^miR-210^. Bar = 500 nm. **b** Western blotting of exosomes for CD63, CD81, CD44, Cytochrome-C and β-actin.

**
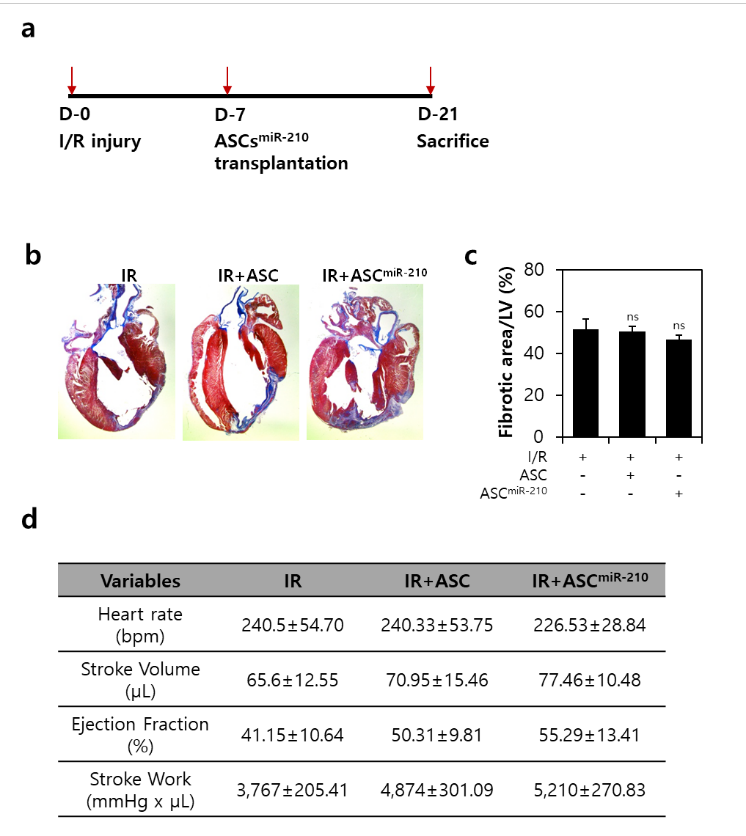
**

**Supplementary Fig. 3 MiR-210-transfected ASCs may also affect on cardiac regeneration, but not significant changes on cardiac fibrosis. a** Schedule for transplantation of ASCs^miR-210^ after IR. After 1 week of I/R injury, ASCs transfected with miR-210 were treated in the border zone and sacrificed at 2 weeks later for various analyzes. **b** Trichrome stain section of I/R heart. **c** Fibrotic area of left ventricles. **d** Measurement of LV function by using Millar catheter after IR and treatment.


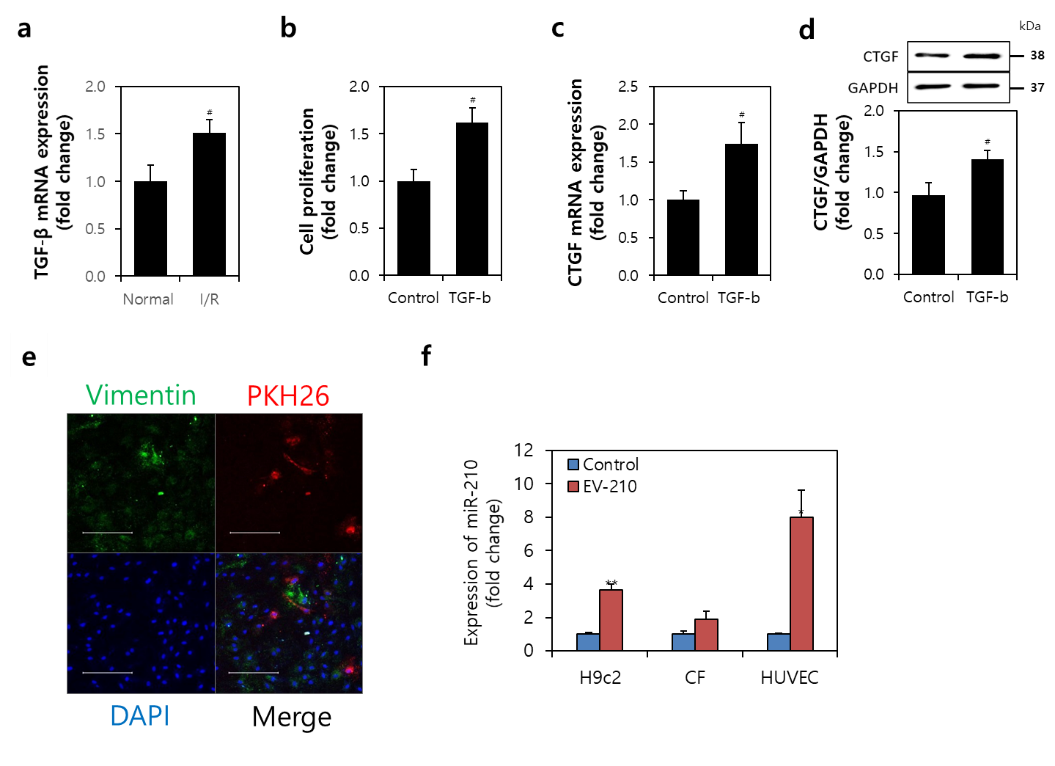


**Supplementary Fig. 4 EV-210 are selectively expressed in cardiac fibroblast exposed to I/R and fibrotic condition. a** Expression of TGF-b mRNA level after I/R injury. Quantitative PCR was used for analysis of normal and I/R injured heart tissue. **b** Measurement of cardiac fibroblast proliferation after TGF-b treatment. Cell proliferation was determined by the colorimetric Cell Counting kit-8 (CCK-8) assay. **c** Messenger RNA expression of CTGF after TGF-b treatment. **d** CTGF expression in TGF-b-stimulated fibroblast. The relative value of western blot band intensity (CTGF/GAPDH) was calculated using an image analyzer. **e** Representative images of immunocytochemical staining for vimentin (green) and PKH26-labeled exosome (Red) of ASCs. Nuclei were counterstained with DAPI (Blue). Bar = 200 μm. **f** Expression level of miR-210 in cardiomyocyte (H9c2), cardiac fibroblast (CF), and endothelial cell (HUVEC) after normal ASC-conditioned medium (Control) or ASC^miR-210^-conditioned medium (EV-210) treatment. CTGF, Connective tissue growth factor; TGF-b, Transforming growth factor beta; GAPDH, Glyceraldehyde 3-phosphate dehydrogenase. *p < 0.05, **p < 0.001. All values are mean ± s.d. Statistical significance was assessed by one-way ANOVA.


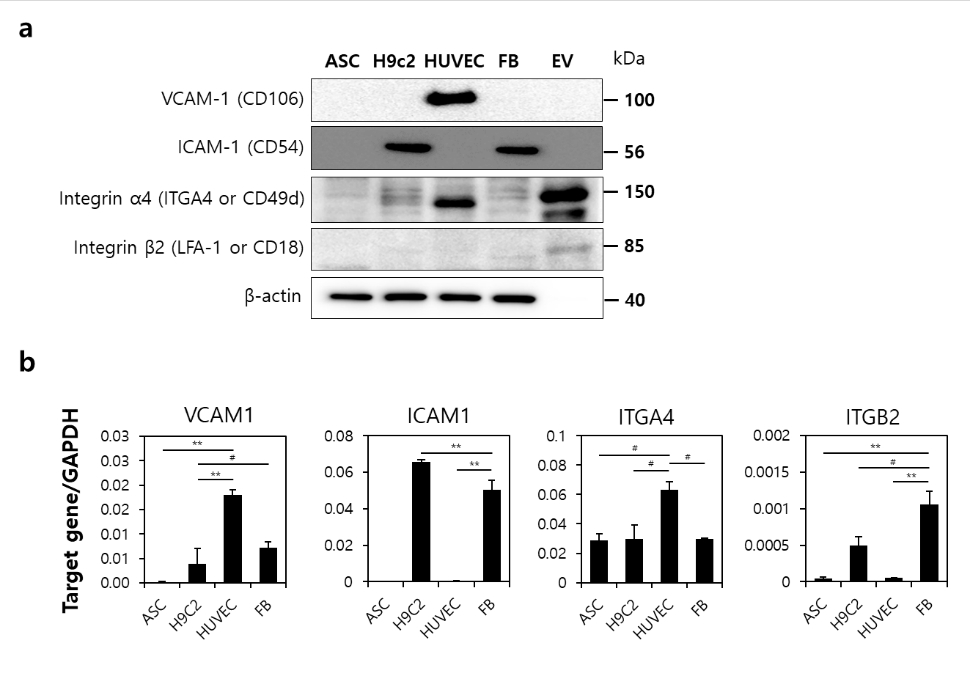


**Supplementary Fig. 5 EVs can connect cell-exosome docking in the heart after ASC transplantation. a** Expression of docking and adhesion proteins in ASC, H9c2, HUVEC, FB, and Exosome (Exo) from ASC. **b** Expression of mRNA level for docking and adhesion molecules. Quantitative PCR was used for analysis of ASC, H9c2, HUVEC, and FB. VCAM1, vascular cell adhesion molecule 1; ICAM1, intercellular adhesion molecule 1; ITGA4, integrin subunit alpha 4; ITGB2, integrin subunit beta 2. * *p* < 0.001 vs. FB, ** *p* < 0.01 vs. FB, ^#^ *p* < 0.05. All values are mean ± s.d. Statistical significance was assessed by one-way ANOVA.

**Supplementary references**

1. He S, Liu P, Jian Z, et al. miR-138 protects cardiomyocytes from hypoxia-induced apoptosis via MLK3/JNK/c-jun pathway. *Biochem Biophys Res Commun*. 2013;441(4):763-769.
2. Sun G, Lu Y, Li Y, et al. miR-19a protects cardiomyocytes from hypoxia/reoxygenation-induced apoptosis via PTEN/PI3K/p-Akt pathway. *Biosci Rep*. 2017;37(6). pii: BSR20170899.
3. Martinez EC, Lilyanna S, Wang P, et al. MicroRNA-31 promotes adverse cardiac remodeling and dysfunction in ischemic heart disease. *J Mol Cell Cardiol*. 2017;112:27-39.
4. Wang X, Ha T, Hu Y, et al. MicroRNA-214 protects against hypoxia/reoxygenation induced cell damage and myocardial ischemia/reperfusion injury via suppression of PTEN and Bim1 expression. *Oncotarget*. 2016;7(52):86926-86936.
5. Yang N, Wu L, Zhao Y, et al. MicroRNA-320 involves in the cardioprotective effect of insulin against myocardial ischemia by targeting survivin. *Cell Biochem Funct*. 2018;36(3):166-171.
6. Zhan L, Lei S, Li W, et al. Suppression of microRNA-142-5p attenuates hypoxia-induced apoptosis through targeting SIRT7. *Biomed Pharmacother*. 2017;94:394-401.
7. Sun X, Zuo H, Liu C, et al. Overexpression of miR-200a protects cardiomyocytes against hypoxia-induced apoptosis by modulating the kelch-like ECH-associated protein 1-nuclear factor erythroid 2-related factor 2 signaling axis. *Int J Mol Med*. 2016;38(4):1303-1311.
8. Wu D, Jiang H, Chen S, et al. Inhibition of microRNA-101 attenuates hypoxia/reoxygenation‑induced apoptosis through induction of autophagy in H9c2 cardiomyocytes. *Mol Med Rep*. 2015;11(5):3988-3994.
9. Li Q, Xie J, Li R, et al. Overexpression of microRNA-99a attenuates heart remodelling and improves cardiac performance after myocardial infarction. *J Cell Mol Med*. 2014;18(5):919-928.
10. Arif M, Pandey R, Alam P, et al. MicroRNA-210-mediated proliferation, survival, and angiogenesis promote cardiac repair post myocardial infarction in rodents. *J Mol Med (Berl)*. 2017;95(12):1369-1385.
11. Fasanaro P, D'Alessandra Y, Di Stefano V, et al. MicroRNA-210 modulates endothelial cell response to hypoxia and inhibits the receptor tyrosine kinase ligand Ephrin-A3. *J Biol Chem*. 2008(23);283:15878-15883.
12. Cheng Y, Liu X, Zhang S, et al. MicroRNA-21 protects against the H2O2-induced injury on cardiac myocytes via its target gene PDCD4. *J Mol Cell Cardiol*. 2009;47(1):5-14.
13. Richart A, Loyer X, Néri T, et al. MicroRNA-21 coordinates human multipotent cardiovascular progenitors therapeutic potential. *Stem Cells*. 2014;32(11):2908-2922.
14. Yang D, Wang J, Xiao M, et al. Role of Mir-155 in Controlling HIF-1α Level and Promoting Endothelial Cell Maturation. *Sci Rep*. 2016;6:35316.
15. Qiao Y, Ma N, Wang X, et al. MiR-483-5p controls angiogenesis in vitro and targets serum response factor. *FEBS Lett*. 2011;585(19):3095-3100.
16. Luo Q, Guo D, Liu G, et al. Exosomes from MiR-126-Overexpressing Adscs Are Therapeutic in Relieving Acute Myocardial Ischaemic Injury. *Cell Physiol Biochem*. 2017;44(6):2105-2116.
